# Chemosensory protein 16 has an immune function and participates in host-pathogen interaction in *Galleria mellonella* infected with *Pseudomonas entomophila*

**DOI:** 10.1080/21505594.2025.2471367

**Published:** 2025-02-28

**Authors:** Jakub Kordaczuk, Michał Sułek, Paweł Mak, Alicja Frączek, Iwona Wojda

**Affiliations:** a Institute of Biological Sciences, Department of Immunobiology, Maria Curie-Sklodowska University, Lublin, Poland; b Faculty of Biochemistry, Biophysics and Biotechnology, Department of Analytical Biochemistry, Jagiellonian University, Kraków, Poland; c Doctoral School of Exact and Natural Sciences, Jagiellonian University, Kraków, Poland

**Keywords:** *Galleria mellonella*, chemosensory protein 16, *Pseudomonas entomophila*, insect immunity, antimicrobial peptides

## Abstract

Chemosensory protein 16 was identified in the hemolymph of *Galleria mellonella* as a protein with an amount increasing after oral infection with 10^3 CFU of *Pseudomonas entomophila*, and decreasing after infection with a higher dose (10^5 CFU) of bacteria. The expression of the CSP16 gene occurred in the fat body and in the gut and correlated with changes in the protein level in the hemolymph. The CSP16 protein inhibited *P. entomophila* growth in the concentration range from 0.15 to 6 nM. Additionally, the CSP16 protein showed bactericidal activity against *P. entomophila, Bacillus thuringiensis*, and *Escherichia coli* in the range of 2–18 μM, but only in the presence of protease inhibitors, otherwise it was degraded by extracellular proteases secreted by *P. entomophila*. We demonstrated that the bactericidal activity of CSP16 was related to its ability to perforate bacterial cellular membranes in a dose-dependent manner. The antimicrobial properties of this protein were also confirmed with the use of Atomic Force Microscopy, which showed significant changes in the topology of different bacterial cell surfaces. Finally, when CSP16 was injected *in vivo* into *G. mellonella* larvae one hour after infection with *P. entomophila*, more survivors were observed at particular time-points. Taking into account its immune properties and putative ability to bind bacteria-derived compounds, the possible function of CSP16 in the host-pathogen interaction is discussed.

## Introduction

Insect immune response requires coordinated action of many proteins. Some of them take part in recognition of infection, while others are involved in the activation of signalling pathways and signal transduction [1,2]. Other proteins/peptides are effector molecules involved directly or indirectly in clearance of infection. The main insect effector molecules are antimicrobial peptides, which are usually small, about 2–10 kDa molecules, but those with higher molecular mass are also known [3]. Most of them are cationic, which allows them to bind to negatively charged microbe membranes, with some exceptions of anionic peptides. Antimicrobial peptides can be divided into linear (e.g. cecropins), cysteine-containing and disulphide-bridged (e.g. defensins), and those with a significant predominance of particular amino acids, i.e. proline-, glycine-, or tryptophan-rich peptides. The mode of their antimicrobial action may differ but most peptides create pores in biological membranes, causing microbial death [4]. More than 20 antimicrobial peptides have been found in larvae of the greater wax moth *Galleria mellonella* [5]. These include cecropins, gallerimycin, galiomycin, proline-rich peptides, anionic peptides, heliocin-like peptides, gloverins, defensins, moricins, and Kazal peptide Pr13a [6–14]. Interestingly, there are increasing numbers of proteins or peptides that have antimicrobial activity but additionally perform other biological functions and are referred to as “moonlighting proteins” [15]. For example, lysozyme (14 kDa protein) is a muramidase enzymatically digesting microbial peptidoglycan [16] and a digestive enzyme in Lepidoptera. This protein can also act non-enzymatically against microorganisms [17–19]. Lysozyme is present constantly in *G. mellonella* larval hemolymph, but its amount can increase after infection [20]. Another moonlighting protein is apolipophorin-III (apoLp-III). This 18 kDa protein is an exchangeable apolipoprotein taking part in lipid transport to fly muscles to support them with an energy source for sustained flight. Therefore, apoLp-III alternates between lipid-bound and lipid-free forms [21]. Furthermore, *G. mellonella* apoLp-III is an important player in insect immunity [22]. Firstly, it is a PRR protein and, secondly, it neutralises bacterial endotoxins, such as lipopolysaccharide (LPS), stimulates the expression of antimicrobial peptide and protein genes, acts synergistically with other hemolymph proteins against invading bacteria, and finally has direct antimicrobial activity itself [23–25]. Another moonlighting molecule is cationic protein 8 (GmCP8) identified in *G. mellonella* larvae. This protein has a molecular mass of above 9 kDa and was found in a few studies as a fungal protease inhibitor (FPI), ISPI-1 (inhibitor of serine proteases-1) with no similarity to any known inhibitor of proteases, and fungal-binding protein-1 (FBP 1). Finally, as mentioned above, GmCP8 was shown to bind to LPS, lipoteichoic acid (LTA), and ß-glucan, which is a necessary prerequisite for the phagocytosis of *E. coli*, *M. luteus*, and *C. albicans*, respectively [26,27]. Further work reported inhibitory activity of GmCP8 toward proteolytic enzymes and activity directed against Gram-positive and Gram-negative bacteria and against fungi [28].

The natural *G. mellonella* pathogens include Gram-positive bacteria like *Bacillus thuringiensis*, Gram-negative bacteria like *Pseudomonas entomophila*, which infect through the oral route, and fungi *Beauveria bassiana* and *Metarhizium anisopliae* infecting through the cuticle. In the body of infected insects, entomopathogens secrete virulence factors directed against host’s immune players [29,30]. Insect pathogens and their hosts constantly undergo antagonistic co-evolution: pathogens improve their virulence mechanisms, while insects improve their defense abilities [31,32].

It has been found that the course of infection of the greater wax moth larvae *G. mellonella* with its natural pathogen *P. entomophila* depends on the route of infection and the applied dose of bacteria [9]. The immune response of insects was dose dependent when the larvae were injected with 10 and 50 *P. entomophila* cells. This means that the expression of immune-stimulated genes and the defense properties of the hemolymph of infected *G. mellonella* larvae were proportional to the dose applied, resulting in differences in survival curves. In contrast, after oral application of the same bacteria in two doses, 10^3^ and 10^5^ CFU, antibacterial activity in the hemolymph of infected larvae was detected only after the application of the lower (10^3^ CFU) dose. However, after force-feeding with the higher dose (10^5^ CFU), the larvae exhibited immune response, i.e. increased expression of immune-relevant genes and increased antibacterial activity of low-molecular weight hemolymph components, as visualised by electrophoresis, but no defense activity was detected in total hemolymph [28]. The chromatographic screening for proteins and peptides whose level changes after infection with *P. entomophila* identified the presence of proline-rich peptide 1 and 2, a cecropin D-like peptide, galiomycin, lysozyme, anionic peptide 1, a defensin-like peptide, and a 27 kDa hemolymph protein. Among them, the expression of the lysozyme-encoding gene and the level of the corresponding protein correlated with antibacterial activity in whole larval hemolymph. However, besides the aforementioned proteins whose level underwent changes after immunisation, we also found chemosensory protein 16 (CSP16) – a putative odorant-binding protein that had not yet been characterised. This led us to hypothesise that this protein may perform defensive functions in *G. mellonella* larvae and may thus be an element of host-pathogen interaction.

## Materials and methods

### Insects, microorganisms, and infection

*Galleria mellonella* (Lepidoptera: Pyralidae) larvae were reared on honeybee nest debris at 28°C and 70% humidity in darkness. Last instar larvae were used for the infection experiments. All microorganisms used in this study and their growth conditions are summarised in Table 1.

**Table 1.** Microorganisms used in this study.

Microorganism	Source and growth conditions
*Bacillus thuringiensis subsp. kurstaki HD1*	Genetic Stock Centre, New Haven, CT; LB 37°C
*Escherichia coli D31*	CGSC5165, Genetic Stock Centre, New Haven, CT; LB 37°C
*Escherichia coli JM83* carrying plasmid *pCH110*	Pharmacia-Amersham; LB 37°C
*Micrococcus luteus*	ATCC 4698; LB 37°C
*Pseudomonas entomophila L48*	Frederic Boccard, CNRS, France; LB 30°C
*Pseudomonas aeruginosa*	ATCC 27853; LB 37°C
*Staphylococcus aureus*	Collection of the Department of Genetic and Microbiology, UMCS, Lublin, Poland; LB 37°C
*Candida albicans*	ATCC 10231; YPD, 37°C

To induce the infection, an overnight *Pseudomonas entomophila* culture was centrifuged (8500 × g), washed with Phosphate Buffered Saline (PBS; 140 mM NaCl, 2.68 mM KCl, 10 mM Na_2_HPO_4_, 1.76 mM KH_2_PO_4_, pH 7.4), and suspended to the desired density in the range from 10^3^ and 10^5^ colony forming units (CFU) per 10 μl of PBS for oral infection by force feeding and 50 cells in 5 µl for hemocelic injection. The number of cells was estimated according to optical density (OD) at 600 nm (for oral infection) and with a cell counter (Muse Cell Analyzer, MERCK Millipore, for intrahemocelic injection), followed by plating the cells and calculation of CFU. As a control, the respective volume of PBS was administered by force feeding (10 µl) or injected (5 µl) into the larval hemocel. Then, the larvae were placed on sterile filter paper in Petri dishes. Larvae that regurgitated the applied suspension were removed from the experiment.

For CSP16 purification, the larvae were immunised with non-pathogenic *Escherichia coli* D31 and *Micrococcus luteus* by pricking their last-but-one proleg with a needle dipped into the pellet obtained by sedimentation of overnight cultures of the bacteria grown in LB medium at 37°C [9]. The infected larvae were kept in well-ventilated plastic boxes at 28°C and fed until further use.

### Hemolymph collection, extraction of low molecular weight components, and RP-HPLC analysis

To obtain whole hemolymph, the larvae were anesthetised by cooling down and surface sterilised with 70% ethanol. The insects were punctured with a sterile scalpel, and hemolymph samples were collected in Eppendorf tubes containing a few crystals of phenylthiourea to prevent melanisation. From each larva, 20 μl of hemolymph was taken. Hemolymph of all larvae from the same group (treated in the same way, 10 individuals per one group in each experiment) was pooled into one Eppendorf tube. The samples were centrifuged for 5 min at 200 × g and then for 10 min at 10,000 × g at 4°C. The cell-free hemolymph was kept at − 20°C for further use.

To obtain methanol extracts, each hemolymph pool was diluted 10 times in a mixture containing nine volumes of methanol, water, and acetic acid (90:9:1; v:v:v). After centrifugation at 20,000 × g for 30 min, precipitated proteins were pelleted, and the upper fraction containing low molecular weight proteins and peptides was carefully collected and lyophilised. The obtained lyophilised extracts were then deprived of lipids before HPLC analysis. In brief, they were resuspended in 0.1% (v/v) trifluoroacetic acid (TFA) in the volume of two thirds of the initial volume (n) of hemolymph (2/3n), and then the same volume of n-hexane was added. After thorough mixing, the samples were centrifuged at 20,000 × g for 15 min at 4°C. The upper fraction containing lipids was removed and a 2/3n volume of ethyl acetate was added to the bottom part, mixed, and centrifuged again. The upper phase was removed while the lipid-free bottom part was transferred to new Eppendorf tubes and freeze-dried. The reversed-phase high pressure liquid chromatography (RP-HPLC) analyses of obtained hemolymph extracts were performed essentially as in our previous work [9].

### Purification of CSP16

The CSP16 protein was isolated from the hemolymph of *G. mellonella* larvae immunised by piercing with a needle dipped in a dense suspension containing a mixture of live *E. coli* and *M. luteus* cells. After 24 hours at 28°C in the dark, the insects were punctured with a scalpel and the hemolymph was collected into chilled Eppendorf tubes containing several phenylthiourea crystals (melanisation inhibitor). 800 µl of immunised hemolymph was mixed with 800 µl of solvent A (0.1% v/v trifluoroacetic acid, TFA), 400 µl of solvent B (0.07% TFA containing 80% acetonitrile, both v/v), and 13 µl of TFA. The mixture was shaken for several minutes and then centrifuged at 20,000 × g for 15 min at room temperature. The pellet was discarded, and the clear supernatant was subjected to RP-HPLC separation using an UltiMate 3000 HPLC apparatus (Thermo, Waltham, MA, USA) and a Discovery Bio Wide Pore C18 4.6 mm × 250 mm column (Sigma-Aldrich, St. Louis, MO, USA). The chromatographic analysis was carried out at 40°C using a biphasic gradient of solvents A and B mentioned above (20% to 67% of solvent B in 45 min). The flow rate was 1 mL/min, and the spectrophotometric detection was performed at 220 and 280 nm. The fraction containing CSP16, eluting at ca. 31 min was collected and subjected to re-chromatography using the same column as above and a linear gradient from 40% to 55% of solvent B in 25 min developed at room temperature. The fraction eluting at 17 min, containing pure CSP16, was collected, freeze-dried, and dissolved in water. The homogeneity and identity of the purified protein were confirmed by SDS-PAGE electrophoresis and N-terminal amino acid sequencing performed using an automatic protein sequencer (PPSQ-31A, Shimadzu, Kyoto, Japan). The concentration of the protein in the solution was determined by the bicinchoninic acid assay (BCA, Sigma-Aldrich, St. Louis, MO, USA) calibrated using bovine serum albumin.

### Tris-Tricine electrophoresis and identification of CSP16

The electrophoresis was conducted in Tris-Tricine SDS-PAGE peptide separating gel. The conditions of sample preparation, electrophoresis, fixing, and staining were described elsewhere [33]. Resolved proteins and peptides were electrotransferred on a PVDF membrane, stained with Coomassie Brilliant Blue-G and visualised using Image Lab software (BioRad) as previously described [9]. In order to identify the protein of interest, the respective band was cut out and sequenced using Edman degradation as described in the previous section.

### Antibacterial activity *in* *vitro* assays

The antibacterial activity of CSP16 and cecropin B (Sigma) was determined as described previously [9]. In every assay, overnight cultures of each bacterial species indicated in the given experiment were refreshed by tenfold dilution in LB and grown for additional 2 h at 120 rpm rotation. The bacteria at the logarithmic growth phase were diluted to OD_600_ of 0.02 in the volume of 1 ml. Then, 20 μl were taken and mixed with 4 μl of the respective polypeptide or water as a control. To investigate the antimicrobial activity of CSP16 in the presence of protease inhibitors, 20 μl of bacteria were taken and mixed with 1 μl of edetic acid (EDTA, Sigma Aldrich; final concentration 0.2 mM), 1 μl of phenylmethylsulfonyl fluoride (PMSF, Sigma Aldrich; final concentration 8.3 mM), and 2 μl of the respective polypeptide or water. The final concentration of the CSP16 peptide mixed with a given microorganism ranged from 2 to 18 µM, as indicated in the appropriate figures, and 7.5 µM for cecropin B. Each sample was immediately divided into two 10 μl parts. One pair (one sample containing bacteria mixed with the polypeptide and one sample containing bacteria mixed with water) was incubated for 60 min at 30°C or 37°C (depending on the microorganism) with shaking and then plated (time 60 min), while the second pair was plated immediately (time 0).

For plating, 900 μl of LB were added to each sample (dilution a: 10^−2^) and, after thorough mixing, 10^−3^ and 10^−4^ (dilutions b and c, respectively) were prepared by taking 100 μl of the previous dilution to 900 μl of LB. Then, 900 μl of each dilution were added to 9 ml LB containing soft (0.7%) agar (v/v) cooled down to 40°C and poured on Petri plates. After the medium had solidified, the plates were incubated at 30°C or 37°C (depending on the microorganism) until colonies were clearly visible (overnight). The results are presented as a percentage of CFU that appeared after plating the polypeptide-containing samples in relation to the respective CFU number obtained for samples without the peptide [9].

For the assay, when CSP16 was present directly in the solid medium, the bacterial suspension of OD_600_ = 0.02 (without CSP16) was diluted to 10^−2^, 10^−3^, and 10^−4^ (as described above), and 900 μl of the respective dilution were poured on solid LB (0.7% v/v agar) plates containing an indicated concentration of CSP16. After overnight incubation at 30°C, the number of CFU was counted and presented as a percentage of CFU growing on plates containing a given concentration of CSP16 in relation to their number on plates without the peptide, which was estimated as 100%.

### Antifungal activity

For testing the activity of CSP16 against *Candida albicans*, an overnight culture of fungi was refreshed by 10-fold dilution in YPD and grown for 5 h in conditions described in Table 1. The fungal suspension was mixed with CSP16. The final OD_600_ of the fungi was 0.0025 and the final concentration of CSP16 was 2 μM in the total volume of 24 μl. In the control, water (or protease inhibitors, respectively, as described above) was added instead of CSP16. Each sample was immediately divided into two 10 μl parts. One pair (one sample containing *C. albicans* mixed with the polypeptide and one sample containing the fungi mixed with water) was incubated at 37°C for 60 min, while the other part was plated directly (time 0). For plating, the mixtures (time 0 and 60 min incubation) were serially diluted 15 and 150 times, and 90 µl of each dilution was mixed with 10 ml of liquid YPD containing soft (0.7%) agar cooled down to about 40°C and poured on Petri dishes. After the medium solidified, the plates were kept at 37°C until colonies appeared (overnight). The number of CFU after mixing the fungi with water (instead of CSP16) was taken as 100% for the respective time points. To investigate the antimicrobial activity of CSP16 in the presence of protease inhibitors, the mixtures (including controls without CSP16) additionally contained EDTA (final concentration 0.2 mM) and PMSF (final concentration 8.3 mM).

### Electrophoretic analysis and bioautography of CSP16

The CSP16 protein in the amount of 0.8 µg after 60 min incubation with water or with the *P. entomophila* suspension of OD_600_ = 0.02, as described above, was loaded on 13.8% gel in sample buffer, and the electrophoresis was conducted according to the Laemmli procedure [34]. The protein was visualised with Coomassie Brilliant Blue R-250. The same samples were loaded on the other gel but the gel after the electrophoresis was washed in Triton X-100 to get rid of SDS. After protein renaturation [35], the gel was overlaid with *P. entomophila* cells suspended in LB with 0.7% agar containing egg white lysozyme to increase the sensitivity of the method. After incubation for 18 h at 30°C, growth inhibition zones were captured using a video image analyser Chemi Doc MP Imaging System (Bio-Rad, Hercules, CA, USA).

### Replica plating

To transfer *P. entomophila* colonies from the solid medium containing the indicated concentration of CSP16 to the medium without the protein, sterile filter paper was placed on the starting surface and gently pressed for 30 s. Then, the paper was transferred to a second Petri dish with LB medium solidified with 0.7% agar, and the appropriate side was pressed again. The plates were incubated overnight at 30°C. The CFU were counted and presented as the average percentage of CFU in relation to the respective controls (without CSP16 or transferred from plates without CSP16, respectively).

#### *In vitro* membrane permeabilisation assay

The membrane permeabilisation assay was performed as described previously [9]. The CSP16 solution was pre-incubated in 20 mM phosphate buffer, pH 6.8, with protease inhibitors: 0.2 mM EDTA and 8.3 mM PMSF in the total volume of 21 µl for 15 min at 37°C. Next, 2 µl of a suspension of the mild-logarithmic phase *E. coli* JM83 cells $\left(5\times 10^{5}\mathrm{CFU}\right)$ carrying the pCH110 plasmid encoding β-galactosidase (Pharmacia-Amersham) were added in the same buffer. The final concentration of CSP16 was 6 µM and 18 µM. After 45-min incubation at 37°C, 20 mM HEPES buffer, pH 7.5, containing 150 mM NaCl (220 µl) and a 50 mM p-nitrophenyl-β-d-galactopyranoside solution (5 µl) were added to the mixture. The samples were incubated for 90 min at 37°C, and the absorbance at 405 nm was measured. Live bacteria incubated with water containing the corresponding concentrations of PMSF and EDTA in the growth medium were used as a negative control (0% perforation), and dead bacteria killed by 5 µM of synthetic cecropin B (Sigma-Aldrich) served as a positive control (100% perforation) [24,36].

#### Isolation of the fat body, RNA extraction, reverse transcription, and qPCR

The fat body and the gut were isolated from anesthetised larvae under ice-cold sterile Ringer’s solution (172 mM KCl, 68 mM NaCl, 5 mM NaHCO_3_, pH 6.1, osmolarity 420 mOsm). Organs from five larvae in each group (in every experiment) were pooled in ice-cold Ringer’s solution in Eppendorf tubes. Then, the liquid was removed and the organs were quickly frozen in liquid nitrogen for at least 10 min and stored at −80°C. Total RNA was isolated from the tissues using a GenElute Mammalian Total RNA Extraction Kit (Sigma), followed by DNase treatment (Turbo DNA-free, Life Technology). Reverse transcription was performed with the use of 1 μg of total RNA and random hexamer primers (High Capacity cDNA Reverse Transcription Kit, Life Technology). Quantitative PCR from the cDNA obtained was performed using a Step One Plus PCR System (Applied Biosystems) as described before [9]. The primers for the ribosomal protein S7e gene (a reference) were published before [37], and the primers for CSP16 were as follows: forward: 5”-TGCGTACATTGCGAGATGAAG-3;‘ reverse: 5’-GCTCGGGTACGTTGACAATCA-3.” The relative gene expression was calculated taking into account the reaction efficiency, which was above 90% for all primers [38].

#### Atomic force microscopy (AFM)

AFM was performed as described before [9]. As indicated in the Results section, the bacteria at the logarithmic growth phase were diluted to OD_600_ of 0.02. Log-phase microorganisms were incubated for 1 h at 30°C or 37°C (depending on the microorganism) with water (control) or with purified CSP16 at the concentration of 2 µM. Both samples contained 8.3 mM PMSF and 0.2 mM EDTA. The total sample volume was 300 µl. Then, 300 µl of 20 mM phosphate buffer, pH 6.8, were added. Next, bacterial pellets were washed twice with 20 mM phosphate buffer, pH 6.8, and twice with non-pyrogenic water. Finally, the microorganisms were suspended in 10 µl of non-pyrogenic water, applied onto the surface of freshly cleaved mica discs, and allowed to dry overnight at room temperature before imaging. The surface of *P. entomophila*, *B. thuringiensis*, and *E. coli* cells transferred onto mica discs was imaged using NanoScope V AFM (Veeco, USA) in the Analytical Laboratory, Faculty of Chemistry, Maria Curie-Skłodowska University, Lublin, Poland. The measurements were carried out in the “Peak Force QMN” operation mode using a silicone tip with a spring constant of 0.4 Nm − 1 (NSG 30, NT-MDT). The results were processed using Nanoscope Analysis vl. 40 (Veeco). Three fields on each mica disc were imaged. The RMS roughness values and adhesion force values were calculated from 60 fields with an area of 70 nm × 70 nm for *P. entomophila* and from 90 fields with an area of 100 nm × 100 nm for *B. thuringiensis* and *E. coli*, measured over the entire microbial cell surface in areas of 500 nm × 500 nm. The section profiles and three-dimensional (3D) images of the cells were generated using WSxW 5.0 software (Nanotec, Spain).

### Detection of protease activity

#### Spectrometric method

The *P. entomophila* suspension (vol. 200 µl) from the logarithmic growth phase in LB medium at 30°C was centrifuged for 5 min at 8,000×g. Four µl of the supernatant containing 3 µg of total protein were taken for the assay. As a positive control, 1 pmol of thermolysin (Sigma, Aldrich) in acetate buffer (10 mM sodium acetate, 5 mM calcium acetate, pH 7.5) in the volume of 4 µl was used. Then, 1 µl of water was added to both samples, and an equal volume of an azocasein solution (5 mg/ml in water) and the samples were incubated for 1 h at 30°C. The reaction of digesting azocasein into azopeptides was stopped by the addition of an equal amount (10 µl) of 5% trichloroacetic acid (TCA) and further incubation for 10 min at room temperature. After this time, the samples were centrifuged at 14,000 × g for 5 min to remove undigested azocasein. Next, 9 µl of 5% NaOH were added to 18 µl of the supernatant, and the absorbance was measured at 450 nm against the blank sample (without the post-culture fluid or thermolysin) on 96-well transparent microplates for low volumes (96 Well Half Area Microplate, Corning, USA) using a Benchmark Plus microplate reader (BioRad). While the protease activity was tested in the presence of serine protease inhibitors and metalloprotease inhibitors, the reaction was performed in the presence of PMSF and EDTA at the concentration described in the Result section.

#### In gel method – zymography

A post-culture medium containing 2 µg of total protein and 0.8 µg of thermolysin (Sigma, Aldrich) in acetate buffer (10 mM sodium acetate, 5 mM calcium acetate) was applied on 10% polyacrylamide gel containing 25 mg of bovine casein per 10 ml of the gel. After the run, the gel was washed at room temperature in 2.5% Triton X-100 for 2 h to remove SDS, changing the solution every 30 min, and for 24 h in 50 mM Tris-HCl, pH 7.5, for protein renaturation and digestion of casein. The undigested casein present in the gel was visualised by placing the gel in an Amido Black solution. The background was removed by washing the gel on a 10% acetic acid solution until clear spots appeared, indicating the presence of casein digested by the proteolytic enzymes in the gel.

### Preparation of survival curves

*G*. *mellonella* larvae were treated as described in the Results section and placed in well-ventilated plastic boxes with filter paper and earwax. The boxes were placed at 28 °C. At the indicated time-points, the numbers of living larvae were calculated. Dead animals were taken out of the boxes. The graph was created with the use of the Kaplan Meier estimator [39].

### Statistical methods

Statistical analysis was performed using Sigma Plot 12.5 (Systat Software Inc., USA). Normality of data was assessed with the use of the Shapiro – Wilk test. Significant differences were established at *p* < 0.05. For comparison of more than two groups, one-way ANOVA followed by post-hoc (Tukey’s) tests was used. Significant differences were established at *p* < 0.05. Values marked with the same letters do not different significantly. For comparison of two groups, except the atomic force microscopy assay, the statistical analysis was performed using the Student’s t-test, and significant differences between samples were established at *p < 0.05, **p < 0.01, and ***p < 0.001. The atomic force microscopy results were analysed using the Mann–Whitney U-test. Significant differences are indicated with *p < 0.05, **p < 0.01, and ***p < 0.001. To determine differences in the Kaplan–Meier survival curve, Log-rank and All Pairwise Multiple Comparison procedures (Holm–Sidak method) were performed.

## Results

### Level of CSP16 in the hemolymph of *G. mellonella* larvae is immune-regulated

**Figure 1.**
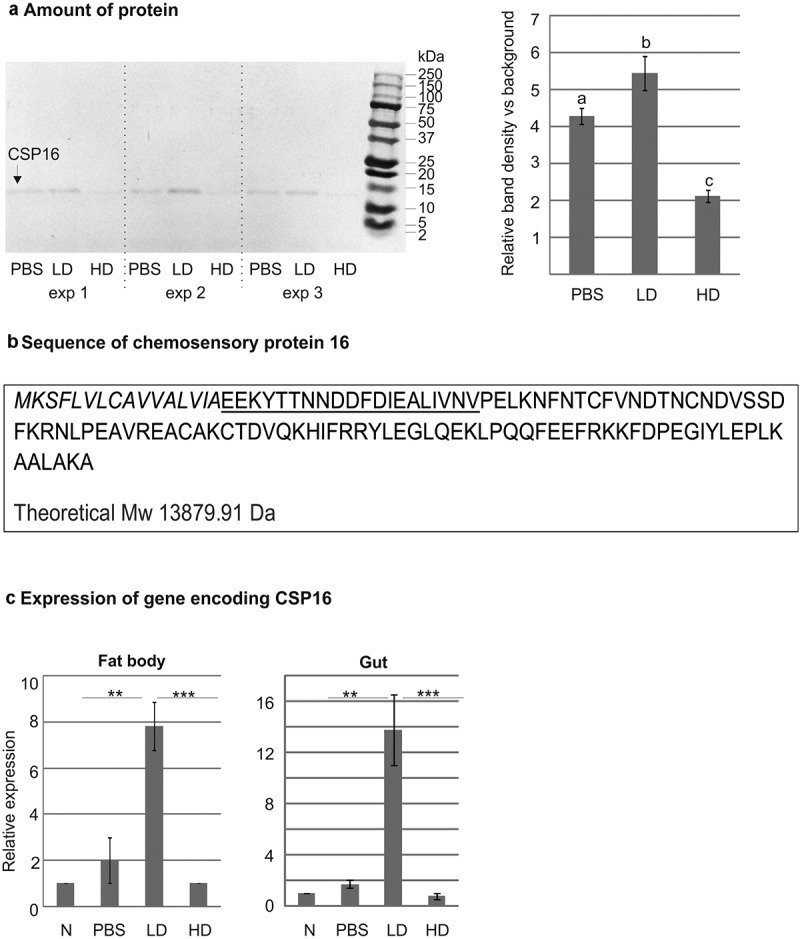
(a) Tris-Tricine electrophoretic analysis of the peptide content in peak number 48 after initial separation by RP-HPLC. Separation of a low molecular fraction of hemolymph obtained from larvae force fed with PBS (PBS), the lower dose (LD, 10^3^ CFU), and the higher dose (HD, 10^5^ CFU) of *P. entomophila* from three experiments. The picture shows the PVDF membrane (left) and the quantitative densitometric analysis of the indicated protein ± SD (right). Different letters show statistically significant differences. The values represent means from three assays (one-way ANOVA, Tukey’s test, *p* < 0.05). An unprocessed and uncropped image is presented in supplementary figure S5. (b) Edman degradation revealed a sequence (underlined) that allowed identifying the protein as chemosensory protein 16 (CSP16). Italic letters show a probable signal sequence. The calculated molecular weight is indicated; it corresponds with the molecular weight of the detected protein. (c) Relative expression of the gene encoding CSP16 in the fat body and gut at 24 h after the oral infection with the lower (LD, 10^3^ CFU in 10 µl) and higher (HD, 10^5^ CFU in 10 µl) doses of *P. entomophila*. As a control, the larvae were also force fed with PBS (PBS); N denotes naive larvae. Mean values ± SD from three assays are shown. Significant differences are indicated (**p < 0.01, ***p < 0.001, Student’s t-test).

In the earlier HPLC analysis of low molecular weight polypeptides of hemolymph collected from *G. mellonella* larvae infected with *P. entomophila*, we found that the relative amount of protein in chromatographic fraction No. 48 increased after the oral administration of 10^3^ cells, but when the dose of bacteria was 10^5^, the peak of the protein was lower (Supplementary Figure S1). We further analysed the content of this fraction using SDS-PAGE electrophoresis. It revealed the presence of a protein with molecular weight around 14 kDa, which was given the working name P48 (protein from fraction No. 48) [40]. Its amount increased after the oral infection with the lower dose of *P. entomophila*; however, when the larvae were orally administered with the higher dose, the amount of the protein decreased even below the control level (Figure 1a).

The Edman degradation of this protein identified an N-terminal amino acid sequence corresponding to putative chemosensory protein 16, whose full amino acid sequence is provided in Figure 1b (the identified sequence is underlined). It is a part of the sequence of protein XP_026762936.2, a product of the LOC113521573 gene, predicted by the automated computational analysis of the *G. mellonella* genome [41]. More details regarding the identification of the CSP16 protein and its similarity to other proteins are provided in Supplementary Table S1.

Since a clear relationship between the amount of CSP16 and the immune status of *G. mellonella* larvae was detected, we independently confirmed this phenomenon by evaluation of the corresponding CSP16 gene expression in the fat body and in the gut of infected larvae. In fact, an about 8- and 15-fold increase in the amount of the transcript was detected in the fat body and in the gut, respectively, but only after the administration of the lower dose of *P. entomophila*, while the relative amounts of the transcripts in larvae infected with the 100-fold higher dose, i.e. 10^5^ CFU, were around the control values (Figure 1c).

### Initial characterisation of CSP16

**Figure 2.**
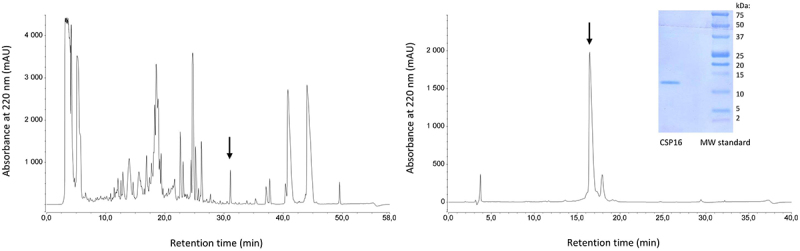
RP-HPLC chromatograms from two successive steps of CSP16 purification. The arrows denote collected fractions, while the insert shows the SDS-PAGE electrophoretic resolution of the purified protein after transfer to PVDF membrane. An unprocessed and uncropped image of the insert is presented in supplementary figure S6.

The CSP16 protein was purified to homogeneity from *G. mellonella* hemolymph in two steps of RP-HPLC chromatography, as described in the Materials and methods section. The pure protein obtained had a molecular mass corresponding to the calculated molecular mass of QEI46814.1 (chemosensory protein 16) and to the protein identified by us and presented in Figure 1a. The purification steps and the image of the final purified protein preparation are presented in Figure 2.

**Figure 3.**
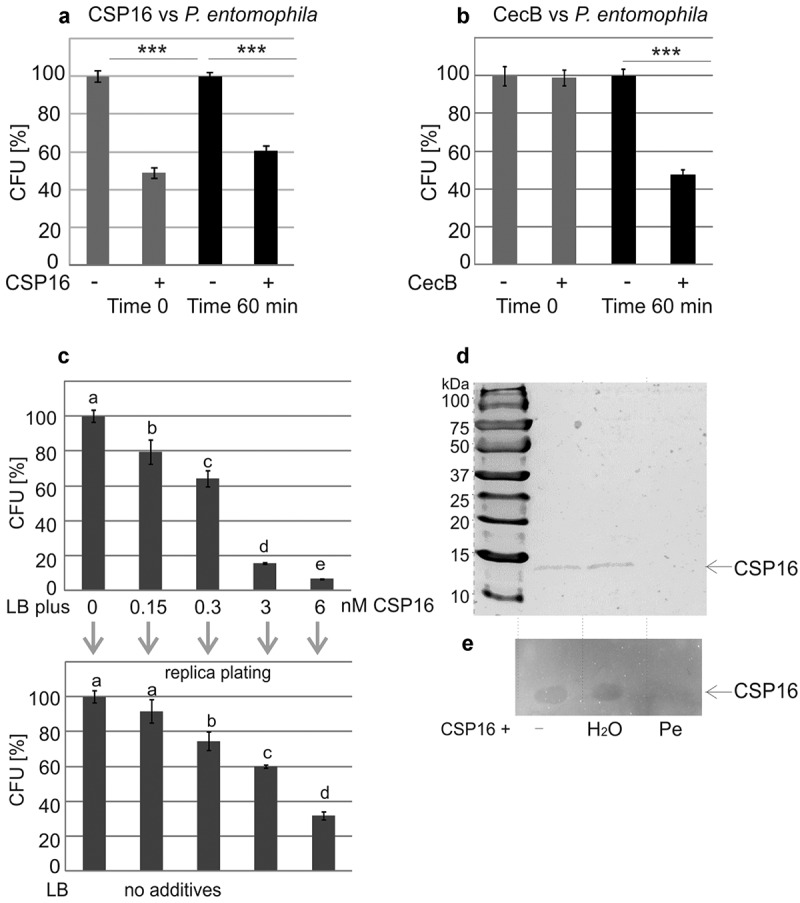
Antibacterial properties of CSP16 (a) and CecB (b) against *P. entomophila*. The assay was performed as described in materials and methods. Mixtures containing 2 μM CSP16 or 7.5 μM Cec B were plated immediately (time 0) or after 60-min incubation at 30° C (time 60). The experiments were performed three times and data from two dilutions were taken for calculation each time; mean values ± SD are shown. Significant differences are indicated (***p < 0.001, Student’s t-test). (c) Growth of *P. entomophila* on plates containing CSP16 at the indicated concentrations (upper part) and after replica plating on LB medium without CSP16 (lower part); the values represent means from three assays; data with different letters show significant differences (one-way ANOVA, Tukey’s test, *p* < 0.05). Part c explains the reduction of the CFU number at time 0 (see a). (d) Detection of CSP16 with no additives (-), after the incubation with water (H_2_O), and with *P. entomophila* (Pe); an unprocessed and uncropped image is presented in supplementary figure S7. The experiment was performed three times. (e) bioautography of the same samples is provided. An unprocessed and uncropped image of the bioautogram is presented in supplementary figure S8. The experiment was performed three times. Parts d and e show that CSP16 disappears after the incubation with *P. entomophila*, which is correlated with the loss of antibacterial properties of the corresponding band.

We checked the antimicrobial properties of CSP16 and noticed that, after its addition to the *P. entomophila* suspension followed by immediate pouring of the mixture onto plates (without incubation, later referred to as time 0), 50% of CFU were observed in relation to the sample without CSP16 (Figure 3a, time 0). When CSP16 and the bacteria were incubated for 60 min at 30°C before pouring, the number of CFU amounted to 60% in relation to the control sample (Figure 3a, time 60). This is an unusual result because a reduction in the number of CFU is typically observed after incubation of bacteria with an antimicrobial compound, as in the case of the action of commercially available cecropin B from *D. melanogaster* (Figure 3b).

One hypothesis was that the reduced number of CFU at time 0 may have been caused by the very fast action of CSP16 towards *P. entomophila*, while the surviving cells may have divided during the 60-min incubation. This hypothesis was ruled out (see Supplementary Figure S2).

Instead, we found that the reduction of CFU at time 0 was caused by the presence of CSP16 in the solid medium, i.e. after pouring of the mixture containing *P. entomophila* and CSP16 on the plates. This was found when the bacteria were seeded on previously prepared plates containing already CSP16 at the final concentration range corresponding to the dilution of CSP16-bacteria mixtures that were plated during previous tests (Figure 3c–top). Since the concentrations of the CSP16 peptide in the LB plates were very low, we attempted to detect some viable bacterial cells that did not proliferate in the presence of CSP16. After replica-plating on the nutrient medium without CSP16, the difference between the number of CFU in the control and the tested samples decreased. The number of CFU detected on the plates with CSP16 changed from about 80%, 65%, 15%, and 5% at the concentration of 0.15 nM, 0.3 nM, 3 nM, and 6 nM to 90%, 75%, 60%, 15%, and 5% after the transfer of the bacteria onto the plates without CSP16, respectively (Figure 3c-bottom). This may indicate the bacteriostatic action of CSP16 at these concentrations.

Further, to explain why the reduction in the number of CFU was not higher at time 60 in comparison to time 0 (and was even lower, see Figure 3a), the mixture containing CSP16 and the bacteria incubated for 60 min at 30°C was subjected to electrophoresis followed by detection of *in situ* antibacterial activity of separated polypeptides. It revealed the disappearance of the respective protein band corresponding to CSP16, resulting in the loss of antibacterial activity of the polypeptide resolved in the gel (Figure 3d,e). This was confirmed by the assay in which CSP16 after 60 min incubation with Pe was re-used for the antibacterial assay and no longer showed activity (See Supplementary Figure S3).

**Figure 4.**
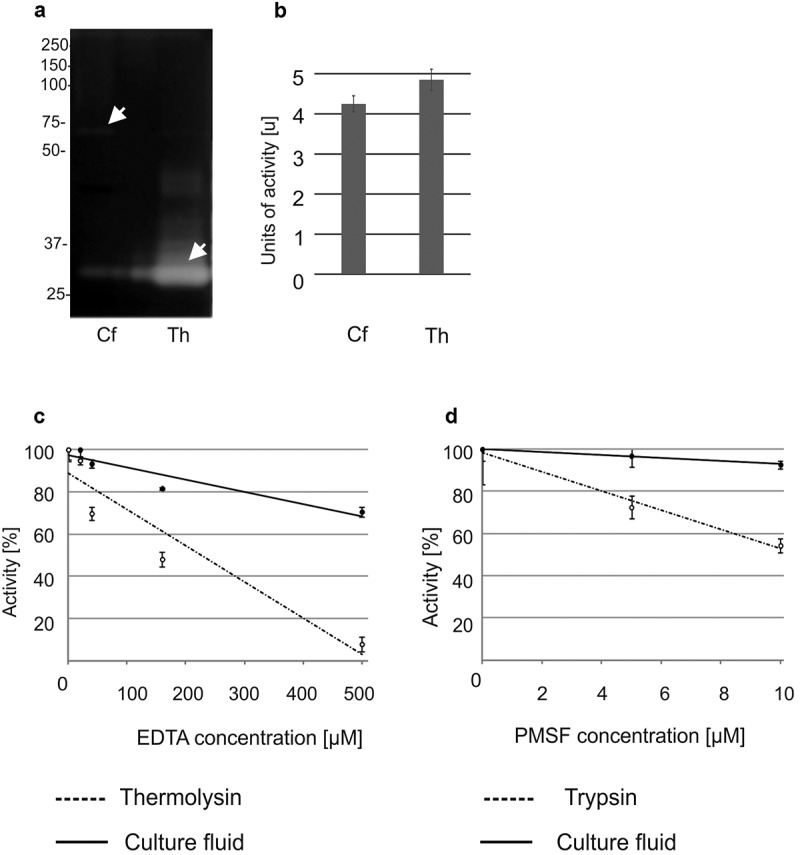
Proteolytic activity of *P. entomophila* culture fluid tested by: (a) zymography, i.e. after prior electrophoretic separation of proteins. The gel was applied to the culture fluid (Cf) containing 2 μg of total protein. The proteolytic activity of the culture fluid was compared to the activity of thermolysin (Th, 0.8 μg were loaded on the gel). Arrows indicate casein digested by a protein above 50 kDa (Cf) and by thermolysin (Th). The experiment was performed three times. An unprocessed and uncropped image is presented in supplementary figure S9. The experiment was performed three times. (b) spectrophotometric method. The culture fluid was obtained and incubated with azocasein according to the method described in materials and methods. Thermolysin was used as a positive control. Both samples showed proteolytic activity. The results are mean values ± SD. One unit was defined as a change in the absorbance of 0.02 at the particular time point in relation to the absorbance value at time 0. The experiments were performed three times. In (c) and (d) the same method was used as in b but in the presence of the indicated amounts of EDTA with thermolysin as a control and PMSF with trypsin as a control, respectively. The results are presented in relation to the activity without the inhibitors, which was estimated as 100%. Mean values ± SD are shown. The experiments were performed three times.

The disappearance of the CSP16 protein in the presence of the bacterial suspension was correlated with proteolytic activity in the *P. entomophila* post-culture medium. Both zymography and the spectroscopic assay detected proteolytic activity appearing in the LB medium in which *P. entomophila* was cultured, which was inhibited by EDTA (inhibitor of metalloproteinases) and slightly inhibited by PMSF (inhibitor of serine proteases, Figure 4).

Summarising, the results presented in this chapter may indicate the following findings:

1. The CSP16 protein inhibits the growth of *P. entomophila* at the concentration range of 0.15–6 nM. The growth is (at least partially) restored when bacteria are transferred to CSP16-free medium.
2. During the 60-min incubation of CSP16 with a suspension of *P. entomophila*, the protein is most likely degraded by proteases secreted by the bacteria. This leads to loss of its antibacterial activity.

### Antimicrobial activity of CSP16

**Figure 5.**
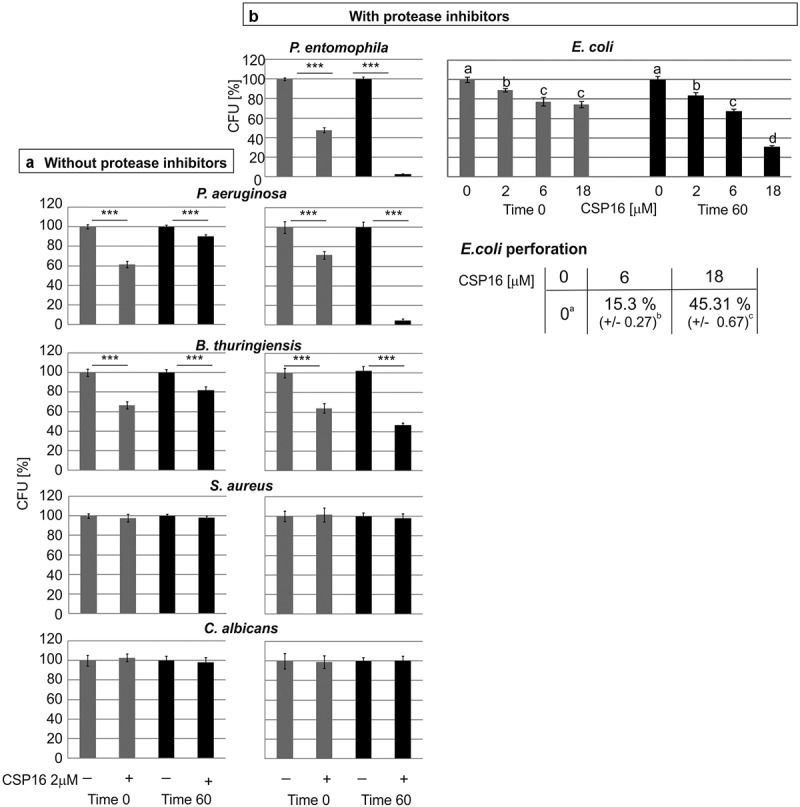
Antimicrobial activity of CSP16 without (a) and in the presence (b) of protease inhibitors (0.2 mM EDTA and 8.3 mM PMSF). In case of *E. coli* JM83 activity of CSP16 at different concentrations was determined in the presence of protease inhibitors. The assay was performed as described in materials and methods with the difference that the controls without CSP16 in b contained protease inhibitors in the same concentration as in samples containing CSP16. The results are shown as a percentage of CFU obtained after incubation with CSP16 in relation to the respective controls at the corresponding time point. Mean values ± SD are shown. The experiments were performed three times and data from two dilutions were taken for calculation each time. Significant differences are indicated; ***p < 0.001 (Student’s t-test). Below, *E. coli* membrane permeabilization was determined by measuring β-galactosidase leakage into the medium after treatment with CSP16. The perforation level of the dead bacteria was assumed as 100% as described in materials and methods. The values represent means from the assays ± SD of three experiments. Statistical significance between the results obtained with two CSP16 concentrations is shown. Data with different letters show significant differences (one-way ANOVA, Tukey’s test, *p* < 0.05).

If the above-presented conclusions are correct, the antimicrobial properties of CSP16 should be revealed when the activity of proteases is inhibited. Indeed, the antimicrobial activity of CSP16 during the 60-min incubation of the protein with *P. entomophila* in the presence of protease inhibitors resulted in an almost complete reduction of the number of CFU (2.5% of CFU in relation to the control without CSP16), while the activity without incubation (time 0) did not change (Figure 5b).

Further, the activity of CSP16 against other microorganisms was tested in the absence and presence of protease inhibitors. We chose another entomopathogenic bacterium, i.e. *Bacillus thuringiensis*, and three opportunistic human pathogens: Gram-negative bacteria *Pseudomonas aeruginosa*, Gram-positive *Staphylococcus aureus*, and yeast *Candida albicans* (see Table 1).

The CSP16 exerted antibacterial activity against *P. aeruginosa* and *B. thuringiensis*. The activity at time 0 was independent of the presence of the protease inhibitors and reduced the number of CFU of both bacterial species to about 60%. In turn, the presence of the protease inhibitors at time 60 min was substantial for the CSP16 activity. Without PMSF and EDTA, the CSP16 activity reduced CFU to about 90% and 80%, while the reduction in the presence of these compounds was about 3% and 45% in the case of *P. aeruginosa* and *B. thuringiensis*, respectively. On the other hand, no microbicidal activity of CSP16 against *S. aureus* and *C. albicans* was detected, irrespective of the presence of the inhibitors (Figure 5).

To investigate the mechanism of the CSP16 action more comprehensively, we checked whether the CSP16 activity resulted in membrane permeabilisation. We used *E. coli* strain JM83 containing a plasmid encoding ß-galactosidase. As in the case of the other microorganisms presented above, the activity of CSP16 against *E. coli* was reduced during the incubation without the protease inhibitors (Supplementary Figure S4). In the presence of EDTA and PMSF, this strain was sensitive to the CSP16 action in a concentration-dependent manner. CSP16 reduced CFU to about 80%, 65%, and 30% at the concentration of 2 µM, 6 µM, and 18 µM, respectively (Figure 5b-right). Independently, we noticed the ability of CSP16 to perforate the *E. coli* membrane, which increased from above 15% at 6 µM to 45% at 18 µM CSP16 (Figure 5b-right).

### CSP16 changes the physical properties of the microbial cellular envelope

**Figure 6.**
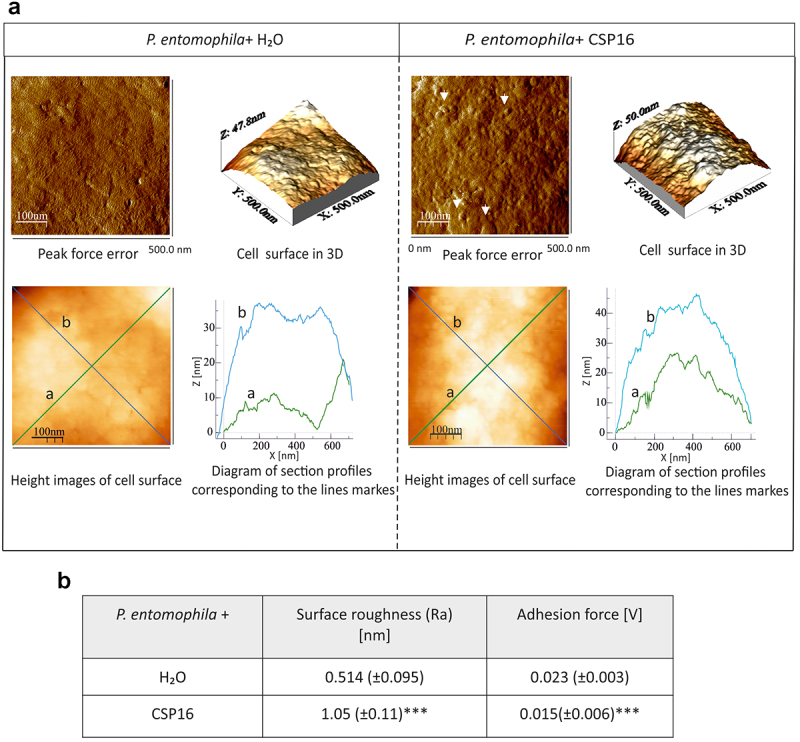
Effect of the CSP16 protein on *P. entomophila* (Pe) cell surface topography. The microorganisms in the logarithmic growth phase were incubated without (water, with protease inhibitors) or with CSP16 (with protease inhibitors) for 1 h. Then, the cells were imaged by AFM. (a) The peak-force-error, three-dimensional, and height images are presented (area of 500 nm × 500 nm), as well as cell surface change profiles measured along lines a and b marked in the height image. The alterations in the cell surface are marked by white arrows. Images taken from three randomly selected areas on the mica disk are presented. (b) The table shows biophysical parameters of the cell surface, such as roughness and adhesion force values. Mean values ± SD taken from three different images are shown. The same number of measurements were performed for each photo within the group; *n* = 60. Significant differences are indicated, ***p < 0.001 (Mann – Whitney U test). The images were taken using nanoscope analysis vl. 40 (Veeco); the section profiles and three-dimensional (3D) images of the cells were generated using WSxW 5.0 sofware (Nanotec, Spain).

We analysed the effect of CSP16 on the topography and biophysical parameters of the microbial surface with the use of Atomic Force Microscopy. Peak force error images (Figure 6a) showed that the surface of *P. entomophila* cells after the exposure to CSP16 became less smooth and more heterogeneous with numerous irregular lumps. This was reflected by the height image of the cell surface, which was also more homogenous in the control cells than in cells exposed to CSP16 (Figure 6a height image). The measurement of section profiles along two lines also showed higher amplitudes in the line course on the surface of cells exposed to CSP16 than in the control cells (Figure 6a, diagram). All these differences were reflected in the measurement of surface roughness, which increased twice under the action of CSP16 (Figure 6b). Also the adhesion of the microscope probe to the surface decreased slightly but significantly, which is also a hallmark of changes in the physical properties of the bacterial surface.

**Figure 7.**
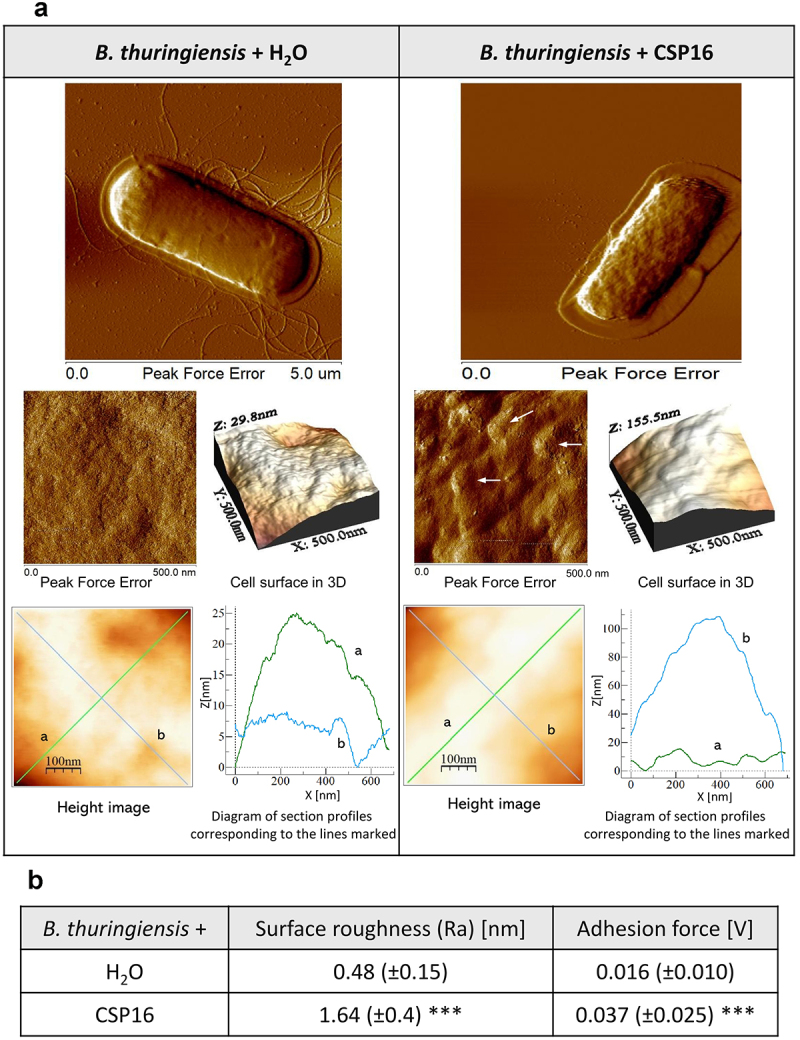
Effect of the CSP16 protein on *B. thuringiensis* (Bt) cell surface topography. The microorganisms in the logarithmic growth phase were incubated without (water, with protease inhibitors) or with CSP16 (with protease inhibitors) for 1 h. Then, the cells were imaged by AFM. (a) The peak-force-error, three-dimensional, and height images are presented (area of 500 nm × 500 nm and 5 μm × 5 μm), as well as cell surface change profiles measured along lines a and b marked in the height image. The alterations in the cell surface are marked by white arrows. Images taken from three randomly selected areas on the mica disk are presented. (b) The table shows biophysical parameters of the cell surface, such as roughness and adhesion force values. Mean values ± SD taken from three different images are shown. The same number of measurements were performed for each photo within the group; *n* = 60. Significant differences are indicated, ***p < 0.001 (Mann–Whitney U-test). The images were taken using nanoscope analysis vl. 40 (Veeco); the section profiles and three-dimensional (3D) images of the cells were generated using WSxW 5.0 sofware (Nanotec, Spain).

**Figure 8.**
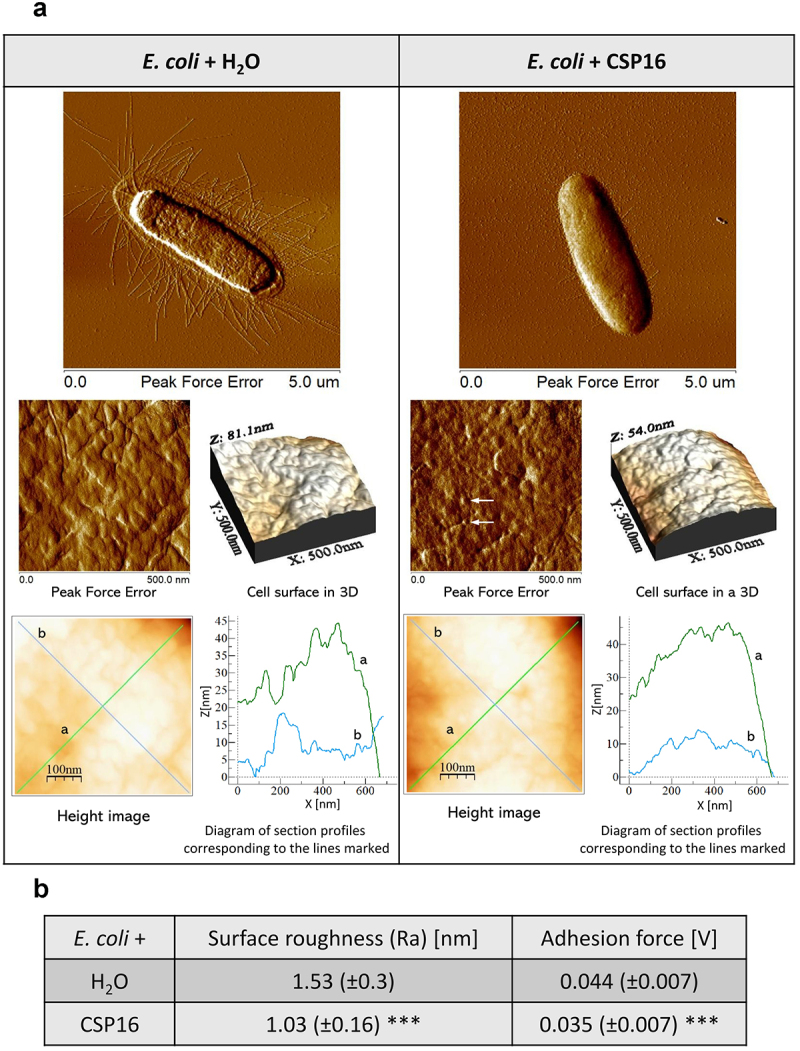
Effect of the CSP16 protein on *E. coli* (Ec) cell surface topography. The microorganisms in the logarithmic growth phase were incubated without (water, with protease inhibitors) or with CSP16 (with protease inhibitors) for 1 h. Then, the cells were imaged by AFM. (a) The peak-force-error, three-dimensional, and height images are presented (area of 500 nm × 500 nm and 5 μm × 5 μm) as well as cell surface change profiles measured along lines a and b marked in the height image. The alterations in the cell surface are marked by white arrows. Images taken from three randomly selected areas on the mica disk are presented. (b) The table shows biophysical parameters of the cell surface, such as roughness and adhesion force values. Mean values ± SD taken from three different images are shown. The same number of measurements were performed for each photo within the group; *n* = 90. Significant differences are indicated, ***p < 0.001 (Mann–Whitney U-test). The images were taken using nanoscope analysis vl. 40 (Veeco); the section profiles and three-dimensional (3D) images of the cells were generated using WSxW 5.0 sofware (Nanotec, Spain).

Increased roughness (threefold) as a result of the CSP16 action was also observed on the surface of *B. thuringiensis* cells. In this case, the appearing lumps seemed to be wider and higher in cells exposed to CSP16 in comparison to the control, and indeed the distance between the lowest and the highest point present on the cellular surface increased about five times (see Figure 7a the Z axis on the 3D structure), which was not the case for *P. entomophila*. Additionally, unlike in the case of *P. entomophila*, the adhesion increased twice in the CSP16-exposed cells (Figure 7b). The bacteria were also smaller and lost their flagella. Finally, we analysed the structure of the *E. coli* cell surface and found loss of fimbriae, a smaller mucous halo, and strong and clear differences in the properties of the cellular surface. The peak force error image showed finer furrows and protrusions in the cells exposed to CSP16 in comparison to the control and a reduced distance between the highest and the lowest point on the surface from 81 to 54 nm (Figure 8a). Also, the profile of the height image lines looked more regular in the case of cells exposed to CSP16, in comparison to those exposed to water, where they were more hackly. Indeed, the roughness value decreased from 1.53 to 1.03 nm, while adherence declined from 0.44 to 0.35 V (Figure 8b).

Summarising, the antibacterial activity of CSP16 against *P. entomophila*, *B. thuringiensis*, and *E. coli* was correlated with changes in the topography of cellular surfaces and their biophysical properties.

### CSP16 changes the survival rate of *G. mellonella* infected with *P. entomophila*

**Figure 9.**
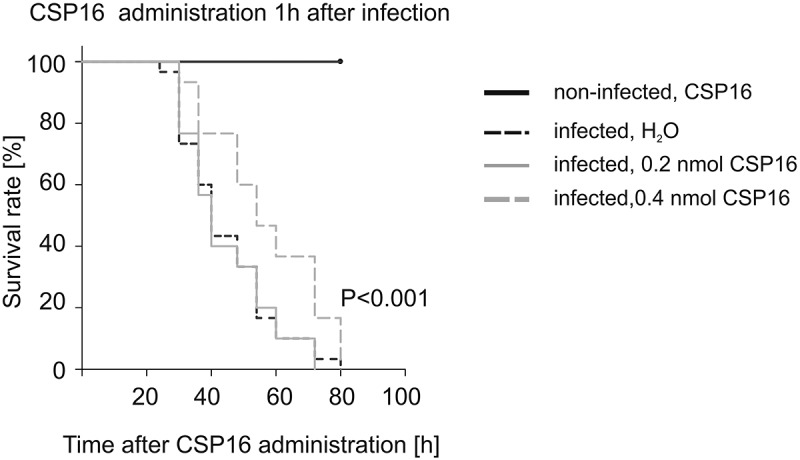
Kaplan–Meier survival curves of *G. mellonella* larvae after intrahemocelic injection with *P. entomophila* and administration of 0.2 nmol and 0.4 nmol of CSP16 (treated larvae) and water (non-treated larvae) 1 h after infection in comparison to the application of 0.4 nmol CSP16. In the experiment, each group comprised 30 larvae. The log rank test followed by the Holm–Sidak method showed statistically significant differences between all presented curves (*p* < 0.05).

To check whether the antimicrobial properties of CSP16 could have physiological significance, we injected this protein to *G. mellonella* larvae in two doses: 0.2 nmol and 0.4 nmol (ca. 3 and 6 micrograms of protein, respectively) one hour after the injection of 50 cells of *P. entomophila*. We noticed an increase in the number of survivors at the particular time-points after the infection in the case of the higher dose applied (Figure 9). This shows the defense abilities of CSP16 *in vivo*. Simultaneously, the protein did not demonstrate toxic properties against the larvae (Figure 9 black solid lane).

## Discussion

We report here the characterisation of the CSP16 protein encoded by the LOC113521573 gene. This ORF was predicted by automatic computational analysis of the *G. mellonella* genomic sequence [41]. The putative uncharacterised protein deduced on the basis of this ORF has been submitted to the NCBI database under accession number XP_0267629.36.2 with the calculated molecular mass 28, 960 Da. The C-terminal part of this putative protein coincides completely with the protein directly submitted to the NCBI database as protein QEI46814.1 with 121 amino acids (NCBI database). Its theoretical molecular weight is 13,879.91 Da, which exactly matches the molecular weight of a protein detected in peak number 48, which was further purified to homogeneity and appeared to be a putative chemosensory protein 16 (CSP16). Chemosensory proteins (CSPs) are part of the olfactory system enabling insects to recognize a broad range of chemicals originating from prey, predators, host plants, conspecifics, and finally mating partners in their environments [42]. The perception of such signals regulates insect behaviour. CSPs take part in the first step of odour perception. External hydrophobic compounds enter the sensillum lymph through epidermal pores and are captured by odorant-binding proteins (OBPs, including pheromones/chemosensory proteins) [43,44]. Then, they are transported with lymph to odorant receptors in the membranes of the dendrites of the olfactory nerves, which trigger a behavior response.

CSP16 was found in *G. mellonella* hemolymph as a protein whose amount increased by about 24% after the infection with entomopathogenic bacteria *P. entomophila* applied orally in the dose of 10^3^ cells per larvae. When the higher dose (10^5^ cells per larvae) was applied, the amount of CSP16 was more than 50% lower in comparison to the control and over 60% lower than its level in the hemolymph of larvae treated with the lower dose. This suggested the participation of this chemosensory protein in the host-pathogen interaction. For example, the drop in the level of CSP16 may have been be a result of the action of pathogen’s virulence factors in several steps, e.g. inhibition of gene expression, faster degradation of its mRNA, inhibition of protein synthesis, or degradation of protein by some pathogen’s virulence factors. We found that the expression of the gene encoding CSP16 in the fat body increased four times after the application of the lower (10^3^ CFU) dose of *P. entomophila*, in comparison to the application of PBS, which was not the case for the 10^5^ dose. The expression observed in the gut of the orally infected larvae was even more pronounced, i.e. there was an up to 13-fold increase in the expression after the application of the lower dose of bacteria (10^3^ CFU), while no significant difference in comparison to the PBS application was observed in the gut after the administration of the higher dose (10^5^). Considering the several to dozen-fold increase in the gene expression, one may expect much higher than 25% differences in the content of CSP16 in the hemolymph of larvae infected with 10^3^ *P. entomophila* cells. However, apart from the regulation of gene expression or mRNA stability, the protein may also be a target for bacterial virulence factors [45,46].

Taking into consideration the interdependence between the relative amounts of transcripts, the CSP16 protein, and the immune status of *G. mellonella*, we undertook characterization of the CSP16 protein in terms of its immune properties. We found that its presence in the LB medium in a concentration ranging from 0.15 nM to 6 nM inhibited the growth of *P. entomophila* cells. This is a low concentration as for the action of antimicrobial peptides, which usually act in micromolar concentrations [47,48]. When the treated bacterial cells were transferred to plates without CSP16, the differences between the control and the CSP16-exposed cells decreased significantly. This means that, in the presence of CSP16, some cells were alive but not able to proliferate, suggesting some bacteriostatic properties of the protein. On the other hand, the difference did not disappear completely, which may indicate killing of the other cells (less likely in our opinion) or may have been caused by the fact that is it much more difficult to replica-plate single cells than a colony (a more likely case, in our opinion). Further studies are needed to confirm these findings.

Our research was focused on the explanation of the reduction of CSP16 antibacterial activity during the incubation of the *P. entomophila* suspension with CSP16. We found that the protein was degraded by the bacterial proteases present in the growth medium. Indeed, the post-culture medium showed protease activity which was inhibited by EDTA and, to a lesser extent, by PMSF, indicating the presence of both metalloproteinases and serine proteases. With the use of the zymography assay, we found that the dominant protease present in the *P. entomophila* post-culture medium had molecular weight above 50 kDa, which is correlated with the mass of the AprA protease, a well-known virulence factor of *P. entomophila* [45]. The susceptibility of CSP16 to the dominant protease present in the culture media is unlikely to involve this particular enzyme. The antibacterial activity of CSP16 was also reduced after the incubation with the other pathogenic bacteria, i.e. *P. aeruginosa* and *B. thuringiensis*. The proteases probably had better access to CSP16 in the liquid medium since CSP16 present in the solid media exerted at least some bacteriostatic effect on the microorganisms.

We then compared the CSP16 antibacterial activity toward the analysed microorganisms in the presence of protease inhibitors, i.e. EDTA and PMSF. It appeared that, after the incubation of CSP16 with *P. entomophila*, *P. aeruginosa*, and *B. thuringiensis*, only 2%, 5%, and 45% colonies remained, respectively. It is also worth to emphasise that the results at time 0 (no incubation) with and without the protease inhibitors were similar, which was an effect of the presence of CSP16 in the solid medium.

Our observations regarding the antimicrobial properties of CSP16 seem to confirm the few reports in this regard. Bianchi et al. [49] reported direct antimicrobial activity of vertebrate OBPs against *C. albicans*, *P. entomophila*, and other bacteria and yeast, suggesting their participation in humoral defense against pathogenic microorganisms. They argued that OBPs may scavenge compounds necessary for their proper growth [49]. Also, Knutelski et al. [50], identified pheromone-binding proteins and odorant-binding proteins by antibacterial radial diffusion assay analysis of RP-HPLC fractions of hemolymph collected from immunised *Rhynchophorus ferrugineus*. They were found among proteins and peptides with known antimicrobial properties, e.g. defensins, cecropin-A1-like peptide, and attacin-B-like protein [50].

The antimicrobial properties of CSP16 and its susceptibility to proteases can explain its increased synthesis during infection as part of the defense mechanism triggered by the host and its decreased level when the infection extends over a threshold, with the prevalence of the pathogen over the infected host. The amount of CSP16 in the hemolymph of infected *G. mellonella* larvae is probably a result of its synthesis and the action of proteases secreted by the intruder, like proteases degrading immune-relevant proteins of the host [45,51,52]. However, a higher bacterial load results in a lower transcription level in comparison to a lower dose, proving that the host-pathogen interaction may occur not only at the level of protein but also directly or indirectly at the nucleic acid level. The direct action may involve the effect of pathogen virulence factors on the stability of mRNA or access of transcription factors to the appropriate DNA region, whereas the indirect effect may involve the impact on certain proteins, e.g. transcription factors [53,54].

We investigated the mechanism of the CSP16 antibacterial activity using two different approaches, both involving the use of protease inhibitors in the assays. Firstly, the ability of membrane perforation was checked with the use of the *E. coli* JM83 strain carrying a plasmid encoding β- galactosidase. Thanks to the fact that CSP16 exhibited activity toward this bacterial strain, we were able to test β-galactosidase leaking from cells treated previously with CSP16. Both the antibacterial activity toward *E. coli* and the activity of β-galactosidase were proportional to the concentrations of CSP16 used for the assay, showing its ability to perforate the bacterial membrane.

Secondly, with the use of AFM, we showed that the exposure of *P. entomophila*, *B. thuringiensis*, and *E. coli* significantly changed the topography and nanomechanical parameters of the cellular surface. In the case of *P. entomophila* and *B. thuringiensis*, the chemosensory protein increased roughness, which was not the case for *E. coli*, where the roughness decreased. A similar effect on the cellular surface, with respect to topography and roughness measurements, was also observed using the AFM technique after the exposure of *P. entomophila* and *B. thuringiensis* to the multifunctional GmCP8 protein, which was reported to act as a multiligand recognition protein and an inhibitor of proteases and to have antimicrobial properties [9,26,27]. On the other hand, the GmCP8 protein also had activity against *C. albicans*, which was not the case for CSP16.

The AFM technique was used before to test the effect of known antimicrobial peptides on the cellular surface. For example, cecropin D purified from *G. mellonella* was shown to affect the nanomechanical properties of *E. coli* JM83, depending on the peptide concentration and the time of bacterial interaction with the antimicrobial compound [24]. Additionally, cecropin A and lysozyme alone or in combination with apoLp- III changed the topography and nanomechanical properties of the *E. coli* surface [24,55]. It is likely that the observed changes in the surface topography are a result of disturbances in the cellular membrane, e.g. its perforation, which was shown in the case of *E. coli*; however, some additional effect like disturbances directly in the cell wall cannot be excluded [56]. Since the level of CSP16 depends on the immune status of *G. mellonella* and the protein exhibits antimicrobial properties, we checked whether the *in vivo* administration thereof in the infected larvae would change their survival curve. Indeed, more alive animals where observed about 50–70 h after infection, which may indicate the *in vivo* action of CSP16. On the other hand, the administration of CSP16 did not extend the lifespan of the larvae, as they all died within 80 h. This may indicate that the injected protein can act *in vivo* a few or a few dozen of hours before being degraded in the hemolymph.

Taking into consideration all the aforementioned properties of CSP16, we may speculate about its role in *G. mellonella* immunity. Some inspiration is provided by a few reports presenting the involvement of chemosensory proteins into recognition of pathogens and induction of insect hygienic behaviours. Recently Shang et al. [57], have reported that chemosensory protein CheA75a in *D. melanogaster* detects the presence of *Metarhizium robertsii* fungal spores on the insect surface and induces behavioural defense by removing the spores from the cuticle. Also, in *Locusta migratoria*, chemosensory protein LmigCSP60 recognises volatiles of its fungal pathogen *Metarhizium acridum*, allowing behavioural avoidance of *M. acridium*-contaminated food [58,59]. Further, it appeared that chemosensory proteins in *Spodoptera frugiperda* could be induced by insecticides and two of them, SfruCSP1 and SfruCSP2, expressed in larval cuticle are able to bind insecticides and their knockdown increases the susceptibility of Spodoptera to three insecticides, namely chlorfenapyr, chlorpyrifos, and indoxacarb [60]. Binding of chemosensory proteins to insecticides was also reported for *Rhaphuma horsfieldii, Plutella xylostella*, and *Conopomorpha sinensis* [61–63].

It seems important that the chemosensory protein 16 had no activity against *S. aureus* and *C. albicans*. It is likely then that *S. aureus* and *C. albicans* do not produce chemical compounds which are recognised by this chemosensory protein, being to a certain extent “invisible” for this protein. Another possibility is that this feature of CSP16 has nothing to do with its ability to bind particular chemicals but, like most antimicrobial peptides, its activity is directed toward certain microorganisms, depending on the physicochemical properties of the microbial surface and the peptide itself [64]. Certainly, whether the ability to bind particular chemicals is necessary for the antimicrobial properties of CSP16 needs to be verified in further research.

**Figure 10.**
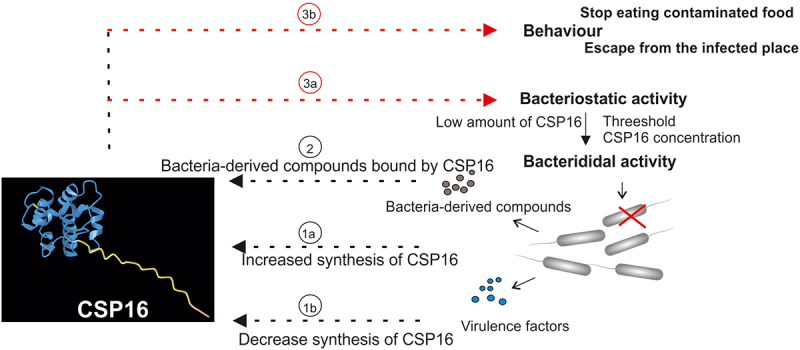
Graphical summary of the discussion section concerning the possible role of CSP16 in the interaction of *G. mellonella* with pathogenic bacteria. Infection triggers the immune response, including up-regulation of CSP16 (1a). On the other hand, proteases secreted by bacteria or some virulence factors contribute to the decrease in the amount of CSP16 (1b). A similar decrease in the level of CSP16 may be an effect of the amount of transcripts or directly the protein. Therefore, the amount of CSP16 is a resultant of both these factors. Volatile compounds secreted by bacteria are bound by CSP16 (2). The binding can result in transport of the odorant to the nerve receptor eliciting changes in behaviour, e.g. cessation of the intake of the contaminated food or escape from the contaminated place (3b). On the other hand, CSP16 directly or after binding of microbe-derived compound attaches to the surface of infecting microorganisms and inhibits its proliferation or kills microorganisms by membrane perforation (3a). The presented possible structure of CSP16 was created on the basis of its primary structure and generated by one of the NCBI blast functions.

It is likely that the CSP16 protein in *G. mellonella* can sense pathogens present in the gut and trigger the “danger” signal, resulting in cessation of the intake of the contaminated food. In the case of infection *via* wounded cuticle, the CSP16 protein may trigger behaviour allowing larvae to escape from the contaminated place. Certainly, we cannot rule out the possibility that binding of CSP16 to intruding microorganisms triggers not only the behavioural defense response but is part of regulatory mechanisms controlling the internal immune response at a level of each individual. Then, the chemical substances produced by pathogens and detected by chemosensory proteins may serve as pathogen-associated molecular patterns (PAMPs) or danger signals DAMPs [65]. The summary of the possible role of CSP16 *in G. mellonella* immunity is presented in Figure 10.

Our findings broadly open the way to further investigations to recognise the role of odorant-binding proteins, and especially chemosensory proteins, in the detection and elimination of intruding microorganisms.

## Supplementary Material

SupplTableS1.docx

## Data Availability

The data that support the findings presented in this article are openly available in Zenodo at https://zenodo.org, reference number 12699269
