# Supplementary material for: Chemosensory protein 16 has an immune function and participates in host-pathogen interaction in Galleria mellonella infected with Pseudomonas entomophila
Source: Virulence. 2025 Feb 28;16(1):2471367. doi: 10.1080/21505594.2025.2471367 (PMC11875508; doi:10.1080/21505594.2025.2471367)
Supplement: SupplTableS1.docx [file KVIR_A_2471367_SM3385.docx]

**Supplementary Table S1.**

**A**. Comparison of CSP16 protein from *G. mellonella* to other proteins with the use of NCBI database (BLAST).

**B**. The amino acid sequence of uncharacterized protein XP_026762936.2, encoded by the gene LOC113521573 and its calculated molecular mass. The underlined letter show amino acids ovelaping with CSP16, which sequence is provided in **C.**

Panel A.

| **Percent of amino acid sequence identity to *G. mellonella* CSP16** | **Protein** | **Length**  **/predicted length** | **Organism** |
| --- | --- | --- | --- |
| 100 % | Uncharacterized protein  XP_026762936 | 257 aa | *Galleria mellonella* |
| 63 % | Putative chemosensory protein  UDM59709 | 122 aa | *Corcyra cephalonica* |
| 60 % | Ejaculatory bulb-specific protein 3-like  XP_059060889 | 121 aa | *Achroia grisella* |
| 53 % | Ejaculatory bulb-specific protein 3  XP_030028678 | 121 aa | *Manduca sexta* |
| 53 % | Chemosensory protein 11  ARO70315 | 122 aa | *Dendrolimus punctatus* |
| 53 % | Insect pheromone-binding family, a10/OS-D domain-containing protein  KAI5643013 | 133 aa | *Phthorimaea operculella* |
| 53 % | Chemosensory protein  AIX97826 | 123 aa | *Cnaphalocrocis medinalis* |
| 52 % | chemosensory protein  APG32546 | 123 aa | *Conogethes pinicolalis* |
| 52 % | hemosensory protein 10  BAV56814 | 120 aa | *Ostrinia furnacalis* |
| 52 % | Chemosensory protein  APG32546 | 123 aa | *Conogethes punctiferalis* |
| 50 % | Chemosensory protein 4  AKT26481 | 123 aa | *Spodoptera exigua* |
| 50 % | Ejaculatory bulb-specific protein 3-like isoform X1  XP_022834083.1 | 130 aa | *Spodoptera litura* |
| 50 % | Ejaculatory bulb-specific protein 3-like isoform X2  XP_022834084 | 123 aa | *Spodoptera litura* |
| 50 % | Chemosensory protein 7 precursor  NP_001037068 | 122 aa | *Bombyx mori* |

Panel B.

**Uncharacterized protein** XP_026762936.2, **LOC113521573**

MNNFEITMKTAFALCVLVAVAVCNAQDTYNPQFDNFNAEEVAGNIRLLKNYGKCFLDQGPCTAEGSDFKRVIPEALQTTCGKCTPKQRVLIRTVVNAFKTKLPDIWEALVQKHDPQGIYKASFDEFLSSQDCVHCEMKSFLVLCAVVALVIAEEKYTTNNDDFDIEALIVNVPELKNFNTCFVNDTNCNDVSSDFKRNLPEAVREACAKCTDVQKHIFRRYLEGLQEKLPQQFEEFRKKFDPEGIYLEPLKAALAKA

**257 aa** This record is predicted by automated computational analysis from a genomic sequence. Calculated molecular mass 28 960 Da (NCBI)

Panel C

**Chemosensory protein 16 QEI46814.1**

**121 aa - direct submission** GeneID:113521573

MKSFLVLCAVVALVIAEEKYTTNNDDFDIEALIVNVPELKNFNTCFVNDTNCNDVSSDFKRNLPEAVREACAKCTDVQKHIFRRYLEGLQEKLPQQFEEFRKKFDPEGIYLEPLKAALAKA

Theoretical Mw 13879. 91 Da
